# Interaction Effects of Child Weight Status and Parental Feeding Practices on Children’s Eating Disorder Symptomatology

**DOI:** 10.3390/nu11102433

**Published:** 2019-10-12

**Authors:** Ricarda Schmidt, Andreas Hiemisch, Wieland Kiess, Anja Hilbert

**Affiliations:** 1 Integrated Research and Treatment Center AdiposityDiseases, Medical Psychology and Medical Sociology, Psychosomatic Medicine and Psychotherapy, Leipzig University Medical Center, Philipp-Rosenthal-Strasse 27, D-04103 Leipzig, Germany; anja.hilbert@medizin.uni-leipzig.de; 2 LIFE Leipzig Research Center for Civilization Diseases, Leipzig University, Philipp-Rosenthal-Strasse 27, D-04103 Leipzig, Germany; andreas.hiemisch@medizin.uni-leipzig.de (A.H.); wieland.kiess@medizin.uni-leipzig.de (W.K.); 3 Department of Pediatrics, Center for Pediatric Research, Hospital for Children and Adolescents, Leipzig University Medical Center, Liebigstrasse 20a, D-04103 Leipzig, Germany

**Keywords:** loss of control eating, eating disorders, feeding strategies, children, population, family

## Abstract

(1) Background: Research on parental feeding practices and non-normative eating behavior including loss of control (LOC) eating and eating disorder psychopathology indicated separate associations of these variables with child weight status, especially in early childhood. This study cross-sectionally examined interaction effects of restriction, monitoring, pressure to eat, and children’s weight status on disordered eating in children aged 8–13 years. (2) Methods: A population-based sample of *N* = 904 children and their mothers completed the Eating Disorder Examination Questionnaire for Children and the Child Feeding Questionnaire. Child anthropometrics were objectively measured. Hierarchical linear and logistic regression analyses were conducted for cross-sectionally predicting global eating disorder psychopathology and recurrent LOC eating by feeding practices and child weight status for younger (8–10 years) and older (11–13 years) ages. (3) Results: Restriction x Child weight status significantly predicted global eating disorder psychopathology in younger children and recurrent LOC eating in older children. Monitoring x Child weight status significantly predicted eating disorder psychopathology in older children. A higher versus lower child weight status was associated with adverse eating behaviors, particularly in children with mothers reporting high restriction and monitoring. (4) Conclusions: Detrimental associations between higher child weight status and child eating disorder symptomatology held especially true for children whose mothers strongly control child food intake.

## 1. Introduction

Controlling feeding practices by parents are well-established parameters in relation to the development and maintenance of children’s weight status, particularly during early and middle childhood [1]. Parental feeding practices are predominantly described as three styles of food-specific parenting, namely, restriction, pressure to eat, and monitoring of children’s eating behavior [2]. Although intended to positively influence child health and weight status, greater parental use of restriction to unhealthy foods increased children’s preferences for these foods and related intake [3,4], while greater parental pressure to eat resulted in adverse reactions to foods and lower food intake in children [5]. Both restriction and pressure to eat were evidenced to be reliable correlates of higher and lower child weight status, respectively, while parental monitoring of children’s eating behavior was not related to child weight status in most previous studies [1,6]. Although there is still ambiguity about the direction of the causal pathway between parental feeding practices and child weight status, recent evidence pointed to a child-effect model, with parents adapting their feeding practices to child weight status [1,7,8]. 

In recent years, research has increasingly focused on identifying the underlying mechanisms for the relationship between parental feeding practices and child weight status. For example, previous studies in early childhood suggested that restrictive feeding patterns inhibit children’s innate ability to self-regulate their eating behavior and to rely less on internal cues of hunger and satiety [9], which could lead to increased food intake and related excess weight [10,11]. Cross-sectional studies indicated substantial associations between specific parental feeding practices and a range of child eating behaviors, as assessed by parent-report through the Child Eating Behavior Questionnaire [12]. Specifically, parental restriction was particularly related to greater food approach behaviors including increased emotional overeating and food responsiveness in children up to 5 years of age [7,13,14,15]. Pressure to eat, however, was consistently associated with food avoidance behaviors in children, including less enjoyment of food, greater slowness in eating, and increased satiety responsiveness and emotional undereating [7,13,14,15]. Two studies in children up to 5 years of age provided inconsistent evidence whether parental monitoring was associated with child eating behaviors [14] or not [13]. 

Food approach and avoidance behaviors, however, provide only a general description of children’s eating behavior without directly indicating the extent of children’s eating disorder symptomatology, such as loss of control (LOC) eating. LOC eating is characterized by eating an objectively or subjectively large amount of food accompanied by a sense of not being able to stop eating. It is highly prevalent in childhood and adolescence [16], is clinically relevant [17,18,19], and can lead to excess weight and eating disorders later in life, at least in vulnerable youth [20,21,22]. In a treatment-seeking sample of *N* = 118 children with overweight and obesity (7–13 years), Matheson et al. [23] showed that greater use of restriction and pressure to eat but not monitoring, as determined via the well-established Child Feeding Questionnaire (CFQ) [2], cross-sectionally predicted the presence of LOC eating, ascertained via clinical interview. A normal-weight control group, however, was not examined. In a large population-based sample of *N* = 2231 adolescents, parental restriction was positively related to dieting and unhealthy weight control behaviors, but not to the presence of binge eating, determined via self-designed items [24]. Relatedly, it was found that the presence of LOC eating episodes, determined through the child version of the Eating Disorder Examination-Questionnaire (ChEDE-Q) [25], was not longitudinally predicted by parental feeding practices, assessed via the CFQ [2], but only by baseline levels of LOC eating and child weight status in a school-based sample including *N* = 424 dyads of 8–12-year-old children and their parents [26]. 

Thus, research findings on the association between parental feeding practices and eating disorder symptomatology in middle and late childhood are rather inconsistent, possibly due to methodological differences across studies, particularly in terms of assessing LOC eating (questionnaire versus interview) and the sample (population-based versus treatment-seeking overweight). In addition, it might be assumed that there are more complex relations worth considering. For example, two studies in *N* = 140 and *N* = 57 5-year-old children indicated that the presence or predisposition to obesity moderated the relationship between parental feeding practices, especially restriction, and disordered eating [4,27]. That is, parental use of restriction was related to adverse consequences only in children with higher versus lower weight status. However, this has not been yet examined in older children, but would be important to identify in order to give specific recommendations for action on feeding practices to parents. In this context, the present study aimed to cross-sectionally examine the predictive value of parental feeding practices and child weight status on children’s eating disorder psychopathology and LOC eating in a large population-based study in German children aged 8 to 13 years. It was hypothesized that restriction, but not pressure to eat and monitoring would positively predict eating disorder psychopathology and LOC eating, particularly in children with higher versus lower weight status. Due to the large age range, the data were separately analyzed for younger (8–10 years) and older (11–13 years) children.

## 2. Materials and Methods 

### 2.1. Procedure

The present study is part of the ongoing “Leipzig Research Center for Civilization Diseases (LIFE)” Child study that started in 2011. One aim of this prospective population-based cohort study is to identify risk factors of childhood obesity and associated mental disorders. For a detailed description of the design and procedures of the LIFE study, see Poulain et al. [28]. For this study, baseline data were used from a sample that was assessed between February 2011 and July 2018. During this period, the Child Feeding Questionnaire (CFQ) [2] and the child version of the Eating Disorder Examination-Questionnaire (ChEDE-Q) [25] were administered to *N* = 1045 mothers and their 8- to 13-year-old children. A total of *n* = 76 (7.3%) and *n* = 65 (6.2%) children was excluded due to missing values or invalid data in the diagnostic items of the ChEDE-Q, respectively (see Data Analytic Plan). No further exclusion criteria were applied. All mothers provided informed consent. Written assent was also obtained from children ≥ 12 years of age. The Ethics Committee of the Medical Faculty of the University of Leipzig, Germany, approved the LIFE study (Reg. No. 264-10-19042010). 

### 2.2. Participants

The final sample consisted of *N* = 904 (52.8% boys) children between 8 and 13 years and their mothers. The majority of mothers was married (*n* = 289 of *n* = 517 with valid data, 55.9%) and of German nationality (*n* = 763 of *n* = 773 with valid data, 98.7%). To assess the families’ socioeconomic status a modified Winkler Index was used which summarizes information about highest educational degree, professional degree, current profession, and household net income [29]. Of those providing valid data (*n* = 877), *n* = 74 (8.4%) families were classified as having low, *n* = 433 (49.4%) medium, and *n* = 370 (42.2%) high socioeconomic status.

The body mass index (BMI, kg/m²) of children was calculated from objectively measured weight and height. Data on child weight and height were based on single measures and assessed by trained research assistants using the electronic personal scale “Seca 701” (Seca Gmbh & Co. KG, Hamburg, Germany) and the calibrated stadiometer “Dr. Keller I” (Längenmesstechnik Limbach GmbH, Limbach-Oberfrohna, Germany). Children’s BMI was transformed into BMI-standard deviation scores (BMI-SDS) using age- and sex-specific reference data from Germany [30]. Accordingly, child weight categories of extreme underweight (BMI-SDS ≤ −1.88), underweight (−1.88 < BMI-SDS ≤ −1.28), normal weight (−1.28 < BMI-SDS < 1.28), overweight (1.28 ≤ BMI-SDS < 1.88), and obesity (≥ 1.88) were determined. For children, the mean BMI-SDS was 0.29 (SD = 1.27), with most children having normal weight (*n* = 605; 67.3%). Maternal BMI, which relied on self-reported weight and height, was available for *n* = 491 mothers. The majority of mothers who provided data on BMI had normal weight (*n* = 252, 51.5%).

### 2.3. Measures

Child Feeding Questionnaire (CFQ). The 31-item CFQ [2,31] was initially developed to assess three parental child feeding practices and four aspects of parental perceptions and concerns about their children’s weight status. In this study, only the three subscales measuring parental feeding practices were analyzed. These feeding practices include restriction (six items), describing the extent to which parents limit their child’s access to foods (e.g., “I intentionally keep some food out of my child’s reach.”), pressure to eat (four items), reflecting parents’ tendency to pressure their children to eat more food (e.g., “I have to be especially careful to make sure my child eats enough.”), and monitoring of eating (three items), depicting the extent to which parents oversee their child’s eating (e.g., “How much do you keep track of the sweet things your child eats?”). All items were rated on a five-point Likert scale expressing agreement (1 = disagree to 5 = agree) or frequency (1 = never to 5 = always), depending on the subscale. Higher subscale mean scores indicate greater manifestation of the respective feeding practice. In the present study, the subscales’ internal consistencies were acceptable to excellent with Cronbach’s α = 0.77 (pressure to eat) and 0.90 (restriction and monitoring). In accordance with recent studies demonstrating increased factorial validity for the modified restriction subscale, the items RST3A and RST3B were separated from the original restriction subscale, as they rather measure parental reward [31]. 

Eating Disorder Examination Questionnaire for Children (ChEDE-Q). The ChEDE-Q [25,32] is a self-report questionnaire for assessing key behavioral features of eating disorders (6 items), including the number of episodes of LOC eating and compensatory behaviors, and children’s specific eating disorder psychopathology (22 items) allocated to four subscales (restraint, eating concern, weight concern, and shape concern). All items refer to the past 28 days and are rated on a seven-point Likert scale (0 = feature was absent to 6 = feature was present every day or to an extreme degree). Based on the four subscales, a global mean score was calculated with higher scores indicating greater eating disorder psychopathology. Internal consistency for the global score in this sample was good (Cronbach’s α = 0.89). The number of LOC eating episodes was based on the item assessing the number of episodes with a sense of having lost control over eating during the past 28 days. In addition to the reporting of the number of LOC eating episodes, recurrent LOC eating defined as the presence of at least 4 LOC eating episodes during the past 28 days (corresponding to a weekly presence of LOC eating) was analyzed categorically (yes versus no), given that the expected mean number of LOC eating episodes will be low and highly skewed in population-based samples and the fact that recurrent LOC eating is associated with greater clinically relevant eating disorder psychopathology than non-recurrent LOC eating [33]. 

### 2.4. Data Analytic Plan

All statistical analyses were performed using IBM^®^ SPSS^®^ Statistics for Windows, version 24.0 (IBM Corp., Armonk, NY, USA). A two-tailed α < 0.05 determined statistical significance. First, ChEDE-Q and CFQ data were checked for missing values, plausibility (e.g., scoring ranges), and outliers. Second, correlation analyses were performed to identify relevant variables to be controlled for in the main analyses. Therefore, the associations of both child (BMI-SDS, age, sex) and family (maternal BMI, age, family socioeconomic status) characteristics with ChEDE-Q and CFQ data were considered. Given that child age, sex, BMI-SDS, and family socioeconomic status showed significant correlations with ChEDE-Q and CFQ data (0.075 ≤ *r* ≤ 0.552, *p* < 0.05), these variables were included as covariates in all analyses. Although maternal BMI was significantly related to ChEDE-Q data (0.092 ≤ *r* ≤ 0.206, *p* < 0.05) and CFQ subscales (0.159 ≤ *r* ≤0.285, *p* < 0.05), maternal BMI was not included in the main analyses due to reduced sample size (*n* = 491), but controlled for in additional analyses when significant prediction effects were found. In case that the addition of maternal BMI changed the results, this was reported. Third, the main analyses were run in order to evaluate the cross-sectional prediction of children’s eating disorder psychopathology and recurrent LOC eating by parental feeding practices and child weight status in hierarchical linear and logistic regression analyses, while controlling for other associated variables described above. In the first step of the hierarchical linear regression of global eating disorder psychopathology, child BMI-SDS, age, sex, and family socioeconomic status were entered. In the second step, CFQ subscales restriction, pressure to eat, and monitoring were included. In the third step, interaction terms of CFQ subscales and child BMI-SDS were added to the model. For the cross-sectional prediction of the presence of recurrent LOC eating using binary logistic regression analysis, the same analytic strategy was used. All predictor variables were mean-centered. Due to the large age range, all analyses were separately conducted for younger (8–10 years) and older (11–13 years) children. *R*² was interpreted as effect size for the goodness-of-fit of the regression model. According to Cohen [34], *R*² = 0.01 indicates a small, *R*² = 0.09 a medium, and *R*² = 0.25 a large effect. A post hoc power analysis was conducted using the software package G*Power version 3.1 (Heinrich-Heine University Düsseldorf, Düsseldorf, Germany). The analysis revealed that the statistical power (1 – β) for the multiple regression analyses (α = 0.05, 10 predictor variables) was 0.99 for detecting a medium to large effect.

## 3. Results

### 3.1. Descriptive Statistics

Descriptive data for the CFQ and ChEDE-Q are age-specifically presented in Table 1. Across the total sample, children reported *M* = 0.74 (*SD* = 1.86) episodes of LOC eating during the past 28 days. Of those reporting any LOC eating episode (*n* = 219, 24.2%), 26.9% (*n* = 59) reported recurrent LOC eating defined as ≥ 4 LOC eating episodes during the past 28 days. Younger and older children did not differ significantly in these variables. However, older children reported significantly higher levels of eating, weight, and shape concern than younger children as well as greater global eating disorder psychopathology. Beyond significantly higher levels of maternal restriction in older versus younger children, there were no other group differences in maternal feeding practices. 

### 3.2. Parental Feeding Practices and Eating Disorder Psychopathology

**Figure 1:**
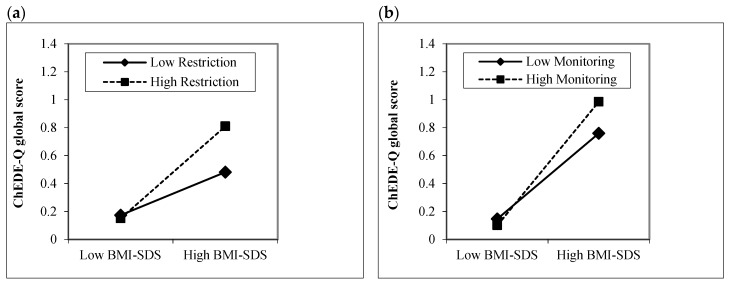
Interaction effect of children’s body mass index-standard deviation score (BMI-SDS) and parental feeding practices (Child Feeding Questionnaire) on children’s global eating disorder psychopathology assessed via the Eating Disorder Examination-Questionnaire for Children (ChEDE-Q): (**a**) Children aged 8–10 years; (**b**) Children aged 11–13 years.

Children aged 8–10 years. In the first step of the linear regression, children’s BMI-SDS (*p* < 0.001), but not age, sex, and socioeconomic status (all *p* ≥ 0.05), significantly positively predicted the ChEDE-Q global score, with medium-to-large-size effect (Table 2). In the second step, parental feeding practices did not significantly add to the amount of variance explained (*p* ≥ 0.05). In the third step, Restriction × BMI-SDS significantly predicted global eating disorder psychopathology with small effect (*p* = 0.001), while the other interaction terms were not significant (*p* ≥ 0.05). As depicted in Figure 1a, children’s BMI-SDS was particularly associated with higher levels of global eating disorder psychopathology in children whose mothers reported high levels of restriction.

Children aged 11–13 years. Among the control variables, children’s BMI-SDS, age, and sex emerged as significant predictors of global eating disorder psychopathology (*p* ≤ 0.006), accounting for 35% of variance indicating a large-size effect (Table 2). A higher BMI-SDS, female sex, and older age were associated with a greater ChEDE-Q global score. In the second step, only maternal restriction significantly predicted children’s eating disorder psychopathology (*p* = 0.034), with small effect. However, after adding interaction terms in the third step, this effect disappeared. Instead, Monitoring × BMI-SDS significantly predicted the ChEDE-Q global score with small effect (*p* = 0.040), indicating that children with higher BMI-SDS reported greater eating disorder psychopathology especially when they had mothers with high levels of monitoring. No other interaction terms were significant (*p* ≥ 0.05, Figure 1b).

### 3.3. Parental Feeding Practices and Recurrent LOC Eating

Children aged 8-10 years. When entered in the first step, child BMI-SDS significantly positively and family’s socioeconomic status negatively predicted (*p* < 0.05) recurrent LOC eating with medium-to-large-sized effect, while no effects were found for child age or sex (*p* ≥ 0.05), see Table 3. Neither the addition of parental feeding practices in the second step nor interaction terms of parental feeding practices and children BMI-SDS significantly improved the prediction effects of recurrent LOC eating (*p* ≥ 0.05). 

**Figure 2:**
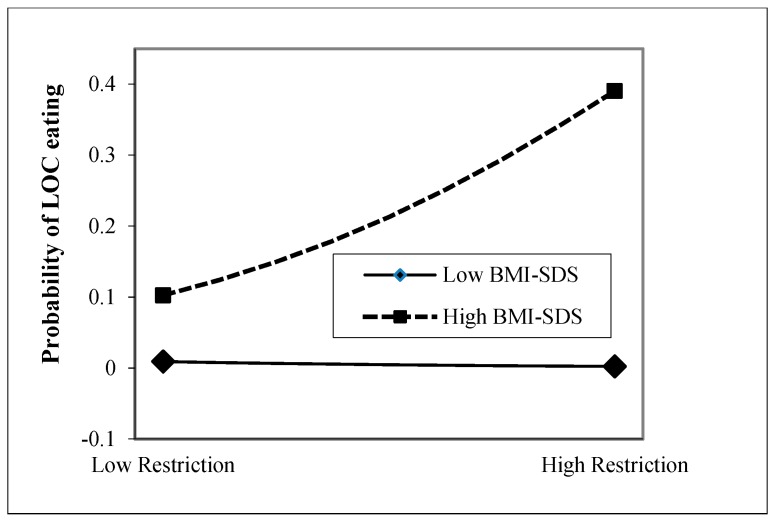
Interaction effect of children’s body mass index-standard deviation score (BMI-SDS) and parental restrictive feeding (Child Feeding Questionnaire) on the presence of recurrent loss of control (LOC) eating assessed via the Eating Disorder Examination-Questionnaire for Children in children aged 11–13 years.

Children aged 11–13 years. In the first step, only children’s BMI-SDS emerged as a significant positive predictor of children’s eating disorder psychopathology with medium effect (*p* < 0.001) among the control variables, as depicted in Table 4. While maternal feeding practices alone did not add any predictive value to the model (*p* ≥ 0.05, step 2), the interaction of Restriction × BMI-SDS significantly predicted the ChEDE-Q global score with small effect (*p* = 0.013), qualifying the main effect of BMI-SDS. As presented in Figure 2, a higher versus lower child BMI-SDS was related to greater odds for reporting recurrent LOC eating in children whose mothers reported high levels of restriction. 

## 4. Discussion

This study sought to determine the associations between specific parental feeding practices, eating disorder psychopathology, and recurrent LOC eating as a form of non-normative eating behavior in a large German population-based sample of 8–13-year-old children and their mothers. The present study revealed that a higher than lower child weight status was particularly related to higher levels of children’s eating disorder psychopathology and greater odds for recurrent LOC eating when maternal use of restriction or monitoring was high. The results thus indicated that the detrimental associations between higher child weight status and greater children’s eating disorder symptomatology may be especially true for children whose mothers are highly controlling children’s food intake. Notably, age-specific effects were revealed: while the combination of greater maternal restriction and higher child weight status was related to children’s eating disorder psychopathology in younger children, it was associated with recurrent LOC eating in older children. In older children, the combined presence of maternal monitoring and higher child weight status was adversely related to children’s eating disorder psychopathology. The study thus adds new insights into the interaction effects of parental feeding practices and child weight status in middle and late childhood, considering that recent studies focused on the single effects of both variables on children’s eating disorder psychopathology so far [23,24,26]. Descriptive analyses revealed a high prevalence of LOC eating (24%), which fits into recently reported prevalence rates ranging between 14% and 27% in samples recruited from the community [26,35]. Of the total sample, 6.9% reported regular occurrence of LOC eating defined as at least four LOC eating episodes during the past 28 days, corresponding to the prevalence rate obtained in a school-based sample of 12–20-year-old youth [33]. 

Although recent studies indicated that highly restrictive feeding practices by parents may be adversely related to child weight status and eating behaviors [6], the use of parental restriction may be considered less serious for children with low weight status. This goes in line with preliminary findings in 5-year-old children addressing the predictive effects of parental feeding practices on child weight trajectories and non-normative eating behaviors [4,27]. The two studies showed that higher levels of parental restriction at age 5 predicted increased weight status and more eating in the absence of hunger at age 7 particularly in children with overweight or those at risk for overweight [4,27]. Though the exact mechanisms of action are unclear, it might be hypothesized that parental restrictive feeding practices in children with higher weight status make them more sensitive to their weight problems and intensify weight control behaviors and dietary restraint [24], thus initiating the vicious circle of LOC eating, at least in a vulnerable proportion of children [36]. Alternatively, since it is assumed that parents adapt their feeding strategies more strongly to deviations in child weight status early in life than vice versa [7,8], a higher child weight status may provoke maternal use of restrictive feeding, with the combination of both conditions increasing children’s disordered eating. Complex bidirectional associations between parental restriction, child weight status, and eating disorder psychopathology are thus to be expected. The fact that the interaction between maternal restriction and child weight status did not predict recurrent LOC eating in the younger age group, but only the level of eating disorder psychopathology, may be due to the lower number of children presenting with weekly LOC eating episodes. 

The result that pressure to eat did not emerge as a predictor adds to the inconsistent findings in middle childhood showing either positive associations with non-normative eating behaviors [23], especially in boys [24], protective effects for eating in the absence of hunger [3], or null associations with LOC eating [26]. In line with recent research [23,24,26], there was no significant main effect for monitoring on eating disorder psychopathology and LOC eating in younger and older children. However, as the first study examining the interactive effects of monitoring and child weight status, it was found that high levels of maternal monitoring were associated with greater eating disorder psychopathology in children with higher than lower weight status, although the interaction effect was smaller compared to that of restriction and weight status. Both monitoring and restriction are feeding practices which are highly controlling in nature. Not surprisingly, these feeding practices have a definite overlap in content as evidenced in a number of studies, with correlations up to *r* = 0.42 [27,37,38,39]. In the present study, the associations between restriction and monitoring were exceptionally high, especially in the older age group (*r* = 0.56 versus *r* = 0.46 in the younger age group), which may help understand the unique result. 

The fact that children’s BMI-SDS was highly associated with eating disorder psychopathology and LOC eating is in line with previous research that documented greater eating disorder psychopathology [40], and higher prevalence of LOC eating in children with overweight and obesity than those with normal weight [16]. Among the covariates, higher child age and female sex were significant predictors of greater global eating disorder psychopathology, which is consistent with a large number of studies [41,42], but they did not predict recurrent LOC eating. In contrast to children’s global eating disorder psychopathology, which subsumes body image concerns that are predominately related to female versus male sex [42], LOC eating is more likely to occur in both girls and boys and across the age range [16,35]. The family’s socio-economic status emerged as a significant predictor of recurrent LOC eating, in accordance with previous literature [33]. 

As a major strength of the present study, a large sample was assessed using internationally established questionnaires with good psychometric properties. Other strengths include the objectively measured height and weight of the children and the inclusion of both boys and girls. In addition, there was a high proportion of children having obesity which allowed to test interaction effects between child weight status and parental feeding practices with sufficient power. At the same time, the sample is not fully representative for Germany as there is a lower prevalence of pediatric obesity [43] and lower rate of mothers with high social status [44] in the general population. Another limitation is the use of a self-report questionnaire instead of an interview-based assessment of eating disorder psychopathology and LOC eating. Finally, for the interpretation of all results, it is of note that the cross-sectional design prohibits causal interpretation.

## 5. Conclusions

In summary, the present study established the moderating effects of child weight status on the cross-sectional relationship between parental restriction and monitoring, global eating disorder psychopathology, and recurrent LOC eating. Given the cross-sectional design of this study, all results should be viewed from a bidirectional perspective: The impact of restriction or monitoring and child weight status on eating disorder psychopathology and LOC eating cannot be interpreted without considering the impact that eating disorder psychopathology and LOC eating may have on parental feeding practices and child weight status. Unfortunately, nothing is known about whether children’s LOC eating, which commonly occurs in secret [19], may elicit parental restriction. Further longitudinal research is warranted to examine causal pathways and delineate the mechanisms contributing to the relationship of parental feeding practices, eating disorder psychopathology, LOC eating, and weight trajectories. This might help to improve early intervention and prevention programs for both eating disorders and obesity.

Clinically, the results particularly indicated the need to broaden the recommendations on parental feeding practices [45], specifically the use of restriction, as restrictive feeding may not necessarily be adversely associated with children’s eating behavior, although longitudinal data are necessary to support this assumption. Instead of providing one guideline on feeding practices to all parents, children’s weight status should be taken into account in this regard. For parents who make intensive use of restrictive feeding, especially when they have children with high weight status, it would be helpful to offer alternative methods of setting limits to children that would enable them to develop appropriate self-control mechanisms.

## Figures and Tables

**Table 1:** Descriptive statistics on sociodemographic and anthropometric data, the Eating Disorder Examination-Questionnaire for Children (ChEDE-Q), and the Child Feeding Questionnaire (CFQ), separately reported for the young and old age group.

	Age Group		Test Statistics
	8–10 years *n* = 380	11–13 years *n* = 524	
**Child**	***M (SD)***	***M (SD)***	
Age, years	9.90 (0.91)	12.03 (0.80)	*t* (750.54) = −36.389 ***
Height SDS	0.25 (0.98)	0.32 (1.00)	*t* (898) = −0.961
Weight SDS	0.25 (1.16)	0.50 (1.26)	*t* (842.94) = −3.017 **
BMI-SDS	0.15 (1.18)	0.39 (1.32)	*t* (855.97) = −2.895 **
	*n* (%)	*n* (%)	
Severe underweight	8 (2.13)	14 (2.68)	χ (4, *N* = 899) = 14.154 **
Underweight	22 (5.85)	34 (6.50)	
Normal weight	278 (73.94)	327 (62.52)	
Overweight	22 (5.85)	47 (8.99)	
Obesity	46 (12.23)	101 (19.31)	
Sex, female	196 (51.58)	231 (44.08)	χ (1, *N* = 904) = 4.965 *
	***M (SD)***	***M (SD)***	
ChEDE-Q Restraint	0.38 (0.88)	0.45 (0.88)	*t* (902) = −1.220
ChEDE-Q Eating Concern	0.41 (0.74)	0.53 (0.84)	*t* (868.50) = −2.422 *
ChEDE-Q Weight Concern	0.66 (1.10)	0.95 (1.31)	*t* (883.76) = −3.663 ***
ChEDE-Q Shape Concern	0.63 (1.11)	1.01 (1.37)	*t* (890.16) = −4.567 ***
ChEDE-Q Global Score	0.52 (0.83)	0.74 (0.98)	*t* (880.79) = −3.593 ***
LOC eating episodes, past 28 days	0.65 (1.68)	0.81 (1.98)	*t* (879.12) = −1.325
Any LOC eating (*n*, %)	85 (22.37)	134 (25.57)	χ (1, *N* = 904) = 1.232
Recurrent LOC eating (*n*, %)	19 (5.00)	40 (7.63)	χ (1, *N* = 904) = 2.504
**Parent**	***M (SD)***	***M (SD*** **)**	
Age, years	39.54 (5.38)	41.22 (5.01)	*t* (748) = −4.403 ***
Winkler index	13.31 (3.44)	12.93 (3.43)	*t* (879) = 1.640
BMI, kg/m²	25.84 (5.74)	26.42 (6.61)	*t* (489) = −1.027
	*n* (%)	*n* (%)	
Underweight	9 (4.11)	5 (1.84)	χ (3, *N* = 491) = 3.389
Normal weight	110 (50.23)	142 (52.21)	
Overweight	59 (26.94)	65 (23.90)	
Obesity	41 (18.72)	60 (22.06)	
	***M (SD)***	***M (SD)***	
CFQ Restriction	2.54 (1.19)	2.77 (1.18)	*t* (900) = −2.823 **
CFQ Pressure to eat	1.77 (0.90)	1.76 (0.86)	*t* (901) = 0.191
CFQ Monitoring	3.52 (1.05)	3.48 (1.04)	*t* (898) = 0.507

Note. BMI: body mass index, LOC: loss of control, *M*: mean, *SD*: standard deviation, SDS: standard deviation score. * *p* < 0.05, ** *p* < 0.01, and *** *p* < 0.001.

**Table 2:** Summary of hierarchical linear regression analyses (full model) for the prediction of global eating disorder psychopathology (ChEDE-Q Global Score) in younger (8–10 years) and older (11–13 years) children.

	8–10 years (*n* = 366)						11–13 years (*n* = 505)					
	*B*	*SE*	β	*p*	∆R²	*p*	*B*	*SE*	β	*p*	∆R²	*p*
**Step 1: Control variables**					0.223	<0.001					0.353	<0.001
Child BMI-SDS	0.242	0.043	0.338	<0.001			0.374	0.036	0.500	<0.001		
Child age	−0.061	0.043	−0.067	0.155			0.121	0.044	0.098	0.006		
Child sex	0.041	0.077	0.024	0.600			0.219	0.071	0.110	0.002		
Socioeconomic status	−0.021	0.012	−0.086	0.076			0.004	0.011	0.016	0.680		
**Step 2: Parental feeding practices**					0.015	0.079					0.012	0.024
Restriction	0.076	0.040	0.107	0.056			0.068	0.041	0.080	0.100		
Pressure to eat	−0.022	0.054	−0.023	0.689			0.018	0.046	0.016	0.397		
Monitoring	0.031	0.045	0.039	0.490			0.045	0.041	0.047	0.282		
**Step 3: Interaction terms**					0.030	0.002					0.020	0.001
Restriction × Child BMI-SDS	0.088	0.031	0.148	0.005			0.036	0.028	0.056	0.206		
Pressure to eat × Child BMI-SDS	−0.059	0.041	−0.079	0.155			−0.053	0.031	−0.066	0.081		
Monitoring × Child BMI-SDS	0.000	0.040	0.000	0.998			0.068	0.033	0.087	0.040		

Note. *B*: unstandardized beta, BMI-SDS: body mass index-standard deviation score, *SE*: standard error.

**Table 3:** Summary of hierarchical logistic regression analyses (full model) for the prediction of recurrent loss of control eating in children aged 8–10 years (*n* = 366).

	*B*	*SE*	Exp(*B*)	CI 95%	*p*	χ²	*df*	*p*	Total *R*²
**Step 1: Control variables**						22.815	4	< 0.001	0.18
Child BMI-SDS	0.590	0.288	1.804	1.026–3.172	0.040				
Child age	−0.460	0.275	0.631	0.368–1.083	0.095				
Child sex	0.154	0.530	1.166	0.413–3.294	0.772				
Socioeconomic status	−0.186	0.080	0.830	0.710–0.971	0.020				
**Step 2: Parental feeding practices**						2.610	3	0.456	0.20
Restriction	−0.067	0.295	0.935	0.525–1.666	0.820				
Pressure to eat	0.404	0.305	1.498	0.824–2.726	0.185				
Monitoring	−0.027	0.306	0.973	0.534–1.773	0.930				
**Step 3: Interaction terms**						3.744	3	0.290	0.23
Restriction × Child BMI-SDS	0.253	0.171	1.288	0.920–1.803	0.140				
Pressure to eat × Child BMI-SDS	−0.163	0.195	0.850	0.580–1.245	0.403				
Monitoring × Child BMI-SDS	−0.228	0.230	0.796	0.507–1.250	0.322				

Note. *B*: unstandardized beta, BMI-SDS: body mass index-standard deviation score, CI 95%: 95% confidence interval, Exp(*B*): estimated odds ratio, *SE*: standard error.

**Table 4:** Summary of hierarchical logistic regression analyses (full model) for the prediction of recurrent loss of control eating in children aged 11–13 years (*n* = 505).

	*B*	*SE*	Exp(*B*)	CI 95%	*p*	χ²	*df*	*p*	Total *R*²
**Step 1: Control variables**						23.172	4	< 0.001	0.11
**Child BMI-SDS**	0.208	0.208	1.231	0.819–1.851	0.317				
**Child age**	0.038	0.229	1.039	0.663–1.628	0.869				
**Child sex**	0.519	0.368	1.681	0.817–3.458	0.158				
**Socioeconomic status**	−0.056	0.053	0.945	0.852–1.049	0.290				
**Step 2: Parental feeding practices**						2.979	3	0.395	0.12
**Restriction**	0.083	0.250	1.087	0.666–1.774	0.739				
**Pressure to eat**	−0.072	0.242	0.930	0.579–1.494	0.764				
**Monitoring**	0.036	0.232	1.037	0.658–1.635	0.876				
**Step 3: Interaction terms**						8.997	3	0.029	0.17
**Restriction × Child BMI-SDS**	0.341	0.137	1.406	1.075–1.840	0.013				
**Pressure to eat × Child BMI-SDS**	−0.066	0.133	0.936	0.722–1.214	0.619				
**Monitoring × Child BMI-SDS**	0.031	0.153	1.032	0.764–1.393	0.838				

Note. B: unstandardized beta, BMI-SDS: body mass index-standard deviation score, CI 95%: 95% confidence interval, Exp(*B*): estimated odds ratio, SE: standard error.
